# Insecticidal Activity of Essential Oil from the Leaves of *Myrciaria floribunda* (Myrtaceae) Against *Sitophilus zeamais* in Stored Maize

**DOI:** 10.3390/plants15081272

**Published:** 2026-04-21

**Authors:** Wevertton Marllon Anselmo, Danilo Gustavo Rodrigues Silva, Fábio Henrique Galdino dos Santos, Ana Carla da Silva, Júlio César Ribeiro De Oliveira Farias De Aguiar, Eduarda Florencio Santos, Gilson José da Silva Gomes Vieira, Camila Soledade de Lira Pimentel, Ana Patrícia Silva de Oliveira, Thiago Henrique Napoleão, Patrícia Maria Guedes Paiva, Patryck Érmerson Monteiro dos Santos, Eurico Eduardo Pinto de Lemos, Daniela Maria do Amaral Ferraz Navarro

**Affiliations:** 1 Department of Fundamental Chemistry, Center for Exact and Natural Sciences, Federal University of Pernambuco (UFPE), Recife 50740-540, Pernambuco, Brazil; wevertton.anselmo@afogados.ifpe.edu.br (W.M.A.); danilo.gustavo@ufpe.br (D.G.R.S.); fabio.henriquegaldino@ufpe.br (F.H.G.d.S.); carla.silva2@ufpe.br (A.C.d.S.); julio.faguiar@ufpe.br (J.C.R.D.O.F.D.A.); eduarda.florencio@ufpe.br (E.F.S.); gilson.vieira@ufpe.br (G.J.d.S.G.V.); camilasoledade_24@hotmail.com (C.S.d.L.P.); 2 Department of Biochemistry, Center for Biosciences, Federal University of Pernambuco (UFPE), Recife 50740-540, Pernambuco, Brazil; anapatricia.oliveira@ufpe.br (A.P.S.d.O.); thiago.napoleao@ufpe.br (T.H.N.); patricia.paiva@ufpe.br (P.M.G.P.); patryck.santos@ufpe.br (P.É.M.d.S.); 3 Center of Agricultural Sciences, Federal University of Alagoas, Maceió 57072-900, Alagoas, Brazil; eurico@ceca.ufal.br

**Keywords:** acetylcholinesterase, amylase, fumigation, *Myrciaria floribunda*, *Sitophilus zeamais*

## Abstract

*Myrciaria floribunda* is a plant found in the Northeast region of Brazil with several insecticidal properties that remain little explored. In this sense, this study aims to investigate the harmful effects of essential oil (EO) from *M. floribunda* leaves against *Sitophilus zeamais*, an important corn pest. The EO was applied in toxicity tests by fumigation, contact, and ingestion, as well as in in vitro assays to evaluate its effects on the activity of the enzymes α-amylase, trypsin, and acetylcholinesterase. Chromatographic analysis of the oil revealed (*E*)-caryophyllene (56.41%), viridiflorol (4.02%) and α-selinene (3.85%) as the main compounds. The essential oil (EO) showed fumigation toxicity, with an LC_50_ of 3.2 μL/L of air, and (*E*)-caryophyllene with an LC_50_ of 3.97 μL/L of air. The EO inhibited insect feeding, altering growth rate and feed conversion efficiency starting at 62.5 μL/g. In this study, an increase in amylase and acetylcholinesterase (AChE) activity was observed. This increase in AChE activity may cause an imbalance in the nervous system, leading to insect death. Thus, the EO of *M. floribunda* may serve as an alternative for the control of *S. zeamais* in stored corn and help prevent significant post-harvest losses for farmers.

## 1. Introduction

Maize (*Zea mays* L.) is one of the most widely cultivated grains worldwide and an essential crop in tropical countries, especially Brazil [1]. Post-harvest losses caused by insect pests remain a major problem, with approximately 20 million tons of grain lost annually [2]. Inadequate pest control has led to the indiscriminate use of chemical insecticides, resulting in insect resistance and increased toxic residues in food [3]. Among maize pests, *Sitophilus zeamais* (Motschulsky) (Coleoptera: Curculionidae) is one of the most damaging species in neotropical regions, attacking grains both in the field and during storage and causing losses [4]. Control is mainly based on fumigant insecticides, which, despite their efficiency, present drawbacks such as human toxicity, residue persistence, and the rapid selection of resistant populations [5,6]. In this context, bioactive plant compounds and secondary metabolites with fumigant properties have gained attention as alternatives for the control of stored-grain pests [7]. These compounds represent a potential substitute for phosphine, the effectiveness of which has declined due to resistance in several pest species [8]. Many plant species produce secondary compounds with insecticidal activity, capable of causing mortality, repellency, growth inhibition, and reduced oviposition—effects often broader and less toxic than those of conventional insecticides [9]. Plant-based products are generally accessible, inexpensive, and environmentally safer, and can be applied as powders, extracts, or essential oils. Essential oils have been widely studied in integrated pest management programs, particularly against *S. zeamais* [10]. The genus *Myrcia* is the largest exclusively Neotropical genus of the family Myrtaceae, which includes species of economic and medicinal importance [11,12]. *Myrciaria floribunda* (H. West ex Willd.) O. Berg is widely distributed throughout Brazil [13]. Some studies have demonstrated the pharmacological and insecticidal potential of *M. floribunda*, with bioactive compounds present in its fruits and essential oil showing anticholinesterasic and insecticidal activity [14,15]. However, its effects on stored-grain pests have not yet been investigated. Therefore, this study aimed to evaluate the insecticidal activity of essential oil extracted from *M. floribunda* leaves against *S. zeamais* in stored maize under laboratory conditions.

## 2. Results

### 2.1. Chemical Composition of Essential Oil (EO) from M. floribunda

The yield of EO obtained by the hydrodistillation of *M. floribunda* leaves was 0.43% (*w*/*w*), and a total of 5.3026 g was extracted. GC-MS analysis revealed 42 volatile compounds in the leaf EO, among which sesquiterpenes were the main group (73.8%) (shown in Table 1). The major chemical compound identified in the essential oil was (*E*)-caryophyllene (56.41%).

### 2.2. Insecticidal Bioassay

#### 2.2.1. Assessment of Toxicity by Contact

The essential oil from *M. floribunda* did not exhibit contact toxicity, as no mortality was observed at any of the concentrations tested.

#### 2.2.2. Assessment of Fumigant Toxicity

**Figure 1:**
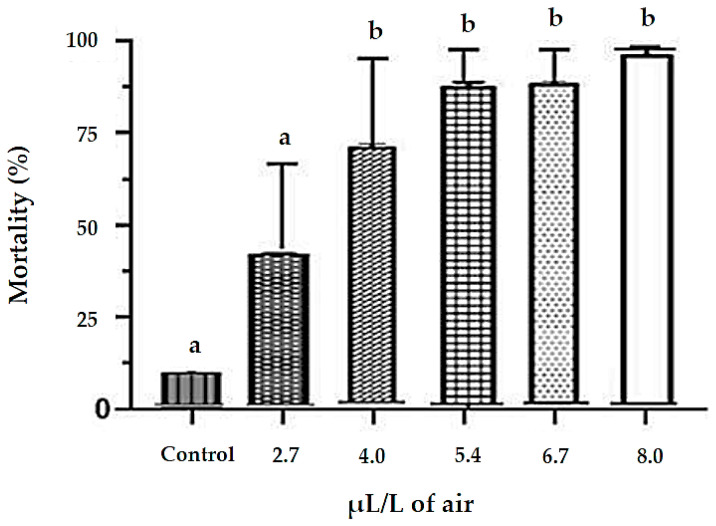
Toxicity of essential oil from *M. floribunda* to *S. zeamais* in fumigation bioassay. Deviations marked with letter a indicate that there is no significant difference (*p* < 0.05) compared to the control. The letter b indicates that there is a difference.

In the fumigant toxicity test, EO from *M. floribunda* increased insect mortality rates as the concentration increased. The statistical analysis showed that treatment solutions at different concentrations differed significantly from the control (Figure 1). The LC_10_, LC_50_, and LC_90_ for the oil and its major constituent (*E*)-caryophyllene are given in Table 2 with respective 95% confidence intervals, fitting the Probit model.

#### 2.2.3. Assessment of Toxicity by Ingestion

**Figure 2:**
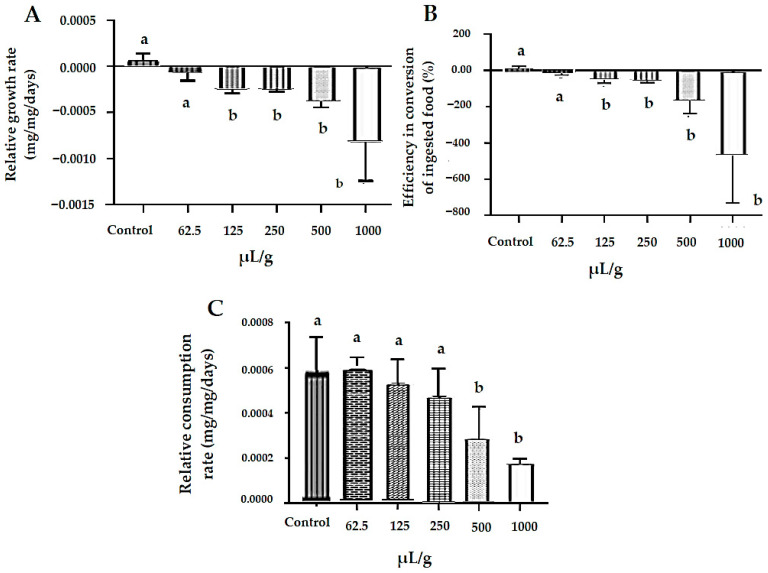
Nutritional indicators of *S. zeamais* adults fed artificial diets containing ethanol (control) or ethanolic solution of *M. floribunda* essential oil (μL of pure oil/g of wheat flour). (**A**) The relative rate of biomass gain indicates the biomass (mg) obtained daily per mg of initial body weight. (**B**) Food conversion efficiency (%) indicates the amount of ingested food converted into biomass by the insects. (**C**) The relative consumption rate indicates the amount of food consumed (mg) per mg of body weight per day. Bars correspond to mean ± SD of three replicates. Deviations marked with letter a indicate that there is no significant difference (*p* < 0.05) compared to the control. The letter b indicates that there is a difference.

**Figure 3:**
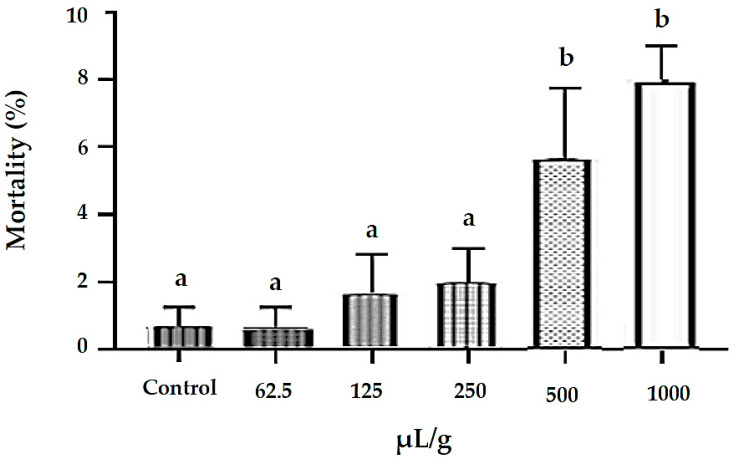
Mortality of *S. zeamais* adults fed artificial diets containing ethanol (control) or ethanolic solution of essential oil from *M. floribunda* (μL of pure oil/g of wheat flour). Bars correspond to mean ± SD of three replicates. Deviations marked with letter a indicate that there is no significant difference (*p* < 0.05) compared to the control. The letter b indicates that there is a difference.

The incorporation of *M. floribunda* essential oil into the insect diet negatively affected nutritional performance, as evidenced by reduced biomass gain (Figure 2A) and impaired food conversion efficiency (Figure 2B), suggesting disruptions in digestive and metabolic processes. Relative consumption rates were significantly reduced at concentrations of 500 and 1000 µL/g (Figure 2C), indicating deterrence or aversion of feeding, which likely contributed to the decreased conversion efficiency. Significant mortality of *S. zeamais* was observed from 62.5 µL/g onward (*p* < 0.05; Figure 3), despite a relatively low deterrence rate (30%), suggesting that mortality was primarily associated with ingestion of toxic oil constituents and possible synergistic interactions among them. Overall, the reduction in food intake combined with decreased food conversion efficiency indicates physiological and metabolic impairment following ingestion of the essential oil, which likely culminated in insect death over time.

#### 2.2.4. Determination of Effects of Essential Oil from *M. floribunda* on Activity of Digestive Enzymes and Acetylcholinesterase

**Figure 4:**
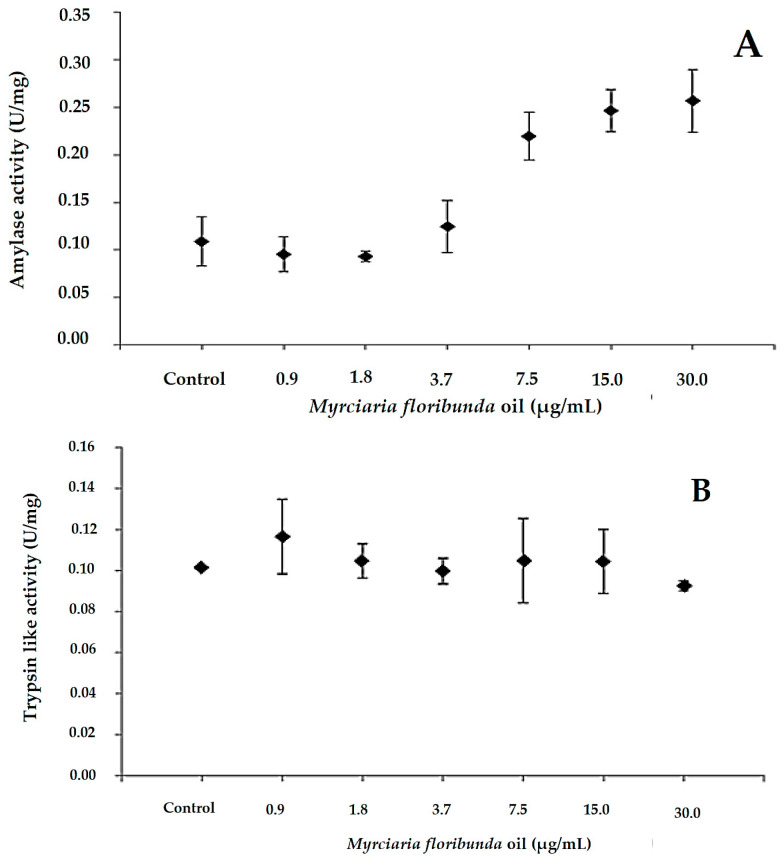

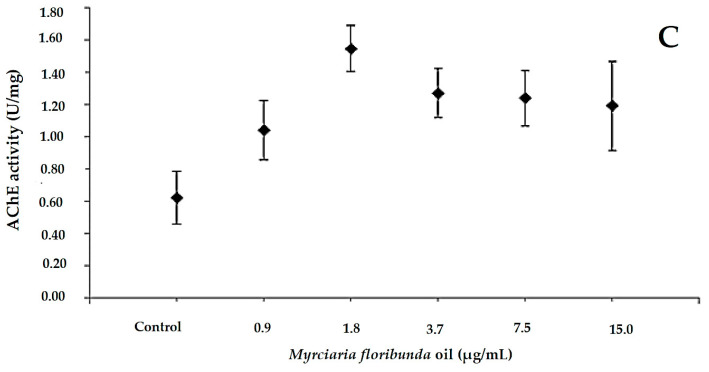
Effects of essential oil from *M. floribunda* on activity of digestive enzymes: α-amylase (**A**), trypsin (**B**), and acetylcholinesterase (**C**).

The EO of *M. floribunda* impacted insect metabolism, influencing the activity of digestive enzymes. Specifically, an increase in amylase activity was observed in the intestinal extract of insects starting at 1.8 µg/mL, and this rate increased with increasing concentration of the essential oil. The increase in amylase activity suggests a possible interference in carbohydrate digestion. Statistically significant differences were found at concentrations of 7.5, 15, and 30 µg/mL of essential oil compared to the control group (Figure 4A). Trypsin enzyme activity was not affected by any of the tested oil concentrations (Figure 4B). However, a decrease in acetylcholinesterase activity was observed in the intestinal extract of adult *S. zeamais* starting at 1.8 µg/mL, followed by saturation of this activity at subsequent oil concentrations (Figure 4C). This result indicates that the essential oil of *M. floribunda* has a neurotoxic effect on the insect. These results indicate that the EO has the ability to modulate the activity of intestinal enzymes in insects, demonstrating its potential in pest management in grains, since its action on digestive enzymes can negatively affect the nervous system of insects and their ability to obtain nutrients.

## 3. Discussion

### 3.1. Chemical Composition

In the present study, (*E*)-caryophyllene was the main component of *M. floribunda* essential oil (EO), in agreement with the results reported by Santos et al. [17]. In contrast, Ramos et al. [18] reported (*E*)-nerolidol as the major constituent (32.4%) with 1,8-cineole at 5.8%, while Tietbohl et al. [15] identified 1,8-cineole as predominant (38.4%) under different extraction conditions. Despite these variations, (*E*)-caryophyllene is frequently reported as a major component in other *Myrciaria* species: Limberger et al. [19] found it as the main constituent in *M. ricardiana* (20.6%) and *M. hatschbachii* (23.3%). Cerqueira et al. [20] reported 10.3–13.2% in *M. salzmannii,* and Pereira-Júnior [21] identified it as predominant in *M. fenestrata* (20.57%), *M. amapensis* (73.12%), *M. caudata* (14.83%), and *M. sylvatica* (45.88%).

### 3.2. Toxicity Tests

#### 3.2.1. Contact

The age of insects is associated with the performance of physiological and biochemical processes in insect metabolism; thus, older insects are described as more susceptible to the toxic effects of essential oils. Studies developed by Nwosu [22], for example, indicate an age-related mortality of over 50% for *S. zeamais* around 36 days, while mortality caused by the insecticide cypermethrin (pyrethroid) was significant only in the 43-to-56-day age range. Therefore, the insects used in the bioassays are outside the age range considered elderly, which makes them more susceptible to insecticide mortality.

Although the EO of *M. floribunda* has in its chemical composition (*E*)-caryophyllene (56.41%) and 1,8-cineole (3.53%), which in the literature are described as lipophilic sesquiterpenes and monoterpenes capable of causing cellular, physiological and behavioral changes [14,15,23,24], no contact mortality effects were observed at any of the concentrations tested. This can be explained by the volatility of the oils or by some difficulty in their penetration into the insect’s cuticle due to resistance mechanisms that involve everything from increased enzymatic metabolism to reduced sensitivity of the chemical compound’s site of action.

#### 3.2.2. Fumigation

The essential oil from *M. floribunda* leaves showed fumigant toxicity against *S. zeamais*, with mortality rates increasing as the concentration increased, and significant differences observed among concentrations, mainly attributed to (*E*)-caryophyllene, α-humulene, and 1,8-cineole. (*E*)-Caryophyllene exhibited strong fumigant activity (LC_50_ = 3.97 μL/L of air) and has also been reported as toxic to *S. oryzae* (LC_50_ = 1.98 μL/L) [21]. This indicates that this major compound is largely responsible for the oil’s activity. 1,8-Cineole, widely distributed in plant species, is well known for its insecticidal effects, and its fumigant toxicity has been reported in essential oils from *Eucalyptus* and *Melaleuca* species against *S. oryzae* (LC_50_ > 20 µL/L of air) [25,26].

#### 3.2.3. Ingestion

Ingestion of artificial food containing *M. floribunda* essential oil (EO) reduced insect weight gain despite increased food consumption, indicating difficulty in nutrient assimilation and suggesting antinutritional effects at 125 μL/g. The EO showed weak feeding deterrence (FDI = 32.27%), indicating detection of the oil without rejection of the food, and suggesting post-ingestion rather than pre-ingestion effects. Similar antinutritional responses without insect mortality were reported for essential oils from *Alpinia purpurata* [27] and *Croton rudolphianus* [28]. Feeding deterrence is not essential for insecticidal activity, as insects may adapt behaviorally to deterrent substances over time [29]. In agreement with these findings, the essential oil from *Myristica fragrans* seeds also affected biomass gain and food consumption in *S. zeamais* [30]. These antinutritional effects may therefore contribute to the control of *S. zeamais* in stored maize.

### 3.3. Activity of Digestive Enzymes and Acetylcholinesterase

Amylases are essential digestive enzymes in insects, particularly in grain pests that feed on starch-rich diets [31,32]. In the present study, amylase activity increased, although the effects of essential oils on this enzyme in *S. zeamais* have not been previously reported. In contrast, inhibitory effects on amylase activity have been described for lectins from *Bauhinia monandra* [33] and in studies with *Aedes aegypti* larvae and *S. zeamais* adults, leading to reduced digestion and antinutritional effects [34,35]. No alteration in trypsin activity was observed, probably due to the starch-rich, protein-poor diet or the absence of protein inhibitors in the essential oil of *M. floribunda*. Although the mechanisms of action of essential oils on AChE are not fully understood, neurotoxic effects have been reported [36,37]. In this study, a low inhibition of AChE was detected, probably induced by insecticidal compounds such as (*E*)-caryophyllene, α-humulene and 1,8-cineole [23]. Studies by Tietbohl et al. [15] report the acetylcholinesterase inhibitory action of essential oil from *M. floribunda* leaves at a concentration of 681 μg/mL, attributed to the action of its main component, 1,8-cineole (38.4%). However, in this study, the percentage of this monoterpene is only 3.8%, which may justify the discreet action of the neurotoxic effect.

In the literature, it is still possible to find studies on the action of essential oils (*Callicarpa rubella* and *Callicarpa macrophylla*) containing (*E*)-caryophyllene in their chemical composition, ranging from 18.0 to 25.2% respectively, with inhibitory activity of acetylcholinesterase (AChE) (LC_50_ = 89.38 ± 4.05 and 221.85 ± 15.32 μg/mL respectively) [38]. Studies by Chaubey [3] showed a 63.89% inhibition of acetylcholinesterase enzyme activity for the β-caryophyllene compound. Similarly, the maximum essential oil concentration of 15 μg/mL tested may not be sufficient to demonstrate a more intense inhibition of AChE activity in S. zeamais. These alterations in the activities of digestive and neural enzymes caused physiological imbalance and mortality in the insects, demonstrating the insecticidal potential of the essential oil of *M. floribunda* and its main constituent, (*E*)-caryophyllene, against *S. zeamais*. These results corroborate the use of this essential oil as a sustainable alternative to synthetic insecticides for the management of *S. zeamais* in stored corn.

## 4. Materials and Methods

### 4.1. Plant Material and Extraction of Essential Oil

Samples of *M. floribunda* in the fruiting stage were collected from the Active Germplasm Bank of Cambuizeiro (BAG—Cambuí), located at the Center for Agricultural Sciences of the Federal University of Alagoas (CECA/UFAL), in Rio Largo, Alagoas, Brazil, (latitude 9°29′45′′ S, longitude 35°49′54′′ W) during the months of July and September 2019, in the morning between 7:00 and 8:00 a.m. There were two collections; however, only the oil from the September collection was evaluated. The sample collection was done randomly, collecting without any selection of plants. In the laboratory, the plant material—branches, leaves, and fruits—was separated, and only the leaves were used and ground in an analytical mill and immediately subjected to the extraction process. Fresh leaves (1100 g) were subjected to hydrodistillation for 3 h using a modified Clevenger apparatus (Le Laborantin, Évreux, France) to obtain the essential oil, which was dried with anhydrous Na_2_SO_4_, stored at 4 °C, and its yield calculated based on fresh weight. The plant material was registered under SisGen number A668DC0.

### 4.2. Analysis of Chemical Composition of Essential Oil

The essential oil’s volatile constituents were analyzed using GC/MS (Agilent 5975C, Agilent Technologies, Palo Alto, CA, USA) and GC (Thermo Trace Ultra, Milan, Italy) following Bezerra-Silva et al. [39] and Da Silva et al. [40]. Compounds were identified by mass spectra and linear retention indices calculated using n-alkane standards (C_9_–C_30_, Sigma Aldrich, Milwaukee, WI, USA), and compared with data from the MassFinder 4, Dr. Hochmuth Scientific Consulting, Hamburg, Germany; NIST08 Mass Spectrum Library (ChemSW Inc., Fairfield, CA, USA); Wiley Registry™ of Mass Spectral Relations 9th Edition (Wiley, Hoboken, NJ, USA) and Adams [16].

### 4.3. Obtainment of Major Constituents (E)-caryophyllene 

(*E*)-caryophyllene (>80%, FCC, FG) was purchased from Sigma-Aldrich, Co., 3050 Spruce Street, St. Louis, MO, USA.

### 4.4. Insecticidal Assay

#### 4.4.1. Insects

A colony of *S. zeamais* was maintained in a B.O.D. incubator-type climate chamber (28 ± 2 °C and 72 ± 10% RH) in 1.5 L containers covered with voile fabric at the UFPE, Brazil. *S. zeamais* adults were fed 150 g of non-transgenic maize kernels per container. Insects aged 7 to 15 days were used for the toxicity bioassays. All toxicity experiments were standardized to be performed in quintuplicate (5 replicates of 5 different concentrations containing 20 insects for each concentration with 3 degrees of freedom). However, preliminary assays were performed in quadruplicate or triplicate to assess at which concentration mortality occurred within the range of 20 to 80%.

#### 4.4.2. Investigation of Toxicity of Essential Oil by Contact

The contact toxicity of the essential oil from *M. floribunda* was assessed using the method described by Lira et al. [27]. Oil solutions were prepared in 1% (*v*/*v*) Tween 80, and concentrations of 20, 83.3, and 166.6 μg/g of insect were used. An aliquot (0.5 μL) of these solutions was applied to the dorsal surface of the thoracic region of the insects using a micropipette. Insects in the negative control group were treated with 0.5 μL of 1% (*v*/*v*) Tween 80. Twenty insects were used in each assay and kept in plastic containers (4.0 × 6.0 cm). The containers were kept in the dark at 28 ± 2°C and 72 ± 10% RH for seven days, followed by the calculation of the mortality rate. The tests were performed in quintuplicate.

#### 4.4.3. Investigation of Fumigant Toxicity of Essential Oil

The fumigant toxicity bioassay was conducted following the protocol described by Chu et al. [41]. The containers were plastic bottles, and the lids were lined with filter paper. The filter paper was previously impregnated with 20 µL of an essential oil solution diluted in ethanol at doses of 2.7, 4, 5.4, 6.7, and 8 µL/L of air. The filter paper in the control group was impregnated with ethanol alone. After a period of 30 s, 20 individuals of *S. zeamais* were placed in each container, and the lids were carefully sealed to form a hermetic chamber. After 24 h of exposure, the insects were transferred to containers with ambient air. The containers were maintained in a B.O.D. type climate chamber (28 ± 2 °C and 72 ± 10% RH), and the mortality rate was recorded after seven days. Each experimental test was repeated in quintuplicate. The same procedure was performed using the major compound in the oil: (*E*)-caryophyllene.

#### 4.4.4. Investigation of Toxicity by Ingestion of Essential Oil

An artificial diet for *S. zeamais* adults was prepared following Lira et al. [27] using autoclaved wheat flour (2 g) mixed with 5 mL of the essential oil solution in 1% Tween 80 at concentrations of 62.5, 125, 250, 500, and 1000 μL/g. The control diet contained wheat flour with 1% Tween 80. Aliquots (200 μL) were placed in Petri dishes (90 × 100 mm) to form feeding discs, dried at 37 °C for 24 h, and weighed. Twenty adults of known weight were transferred per dish, with four replicates per treatment. After seven days at 28 ± 2 °C in the dark, mortality and weights of insects and discs were recorded. Feeding deterrence and nutritional parameters were calculated from ingestion assays. The feeding deterrence index (FDI) was calculated, and the Diets were classified according to Liu et al. [42]. Relative consumption rate (RCR), biomass gain rate (BGR), and food conversion efficiency (FCE, %) were calculated as described by Xie et al. [43].

#### 4.4.5. Preparation of Extract of the Intestine of *S. zeamais* Adults

Intestinal extracts from *S. zeamais* adults were prepared following the protocol described by Napoleão et al. [44]. Adults were removed from the colony and immobilized by cooling at 4 °C for 10 min. The intestines were dissected using a needle, pulling the extremity of the abdomen after removing the elytra. The dissected intestines were kept in an ice bath. Subsequently, 50 intestines were homogenized with 1 mL of a buffer solution (0.1 M sodium acetate, pH 5.5 or 0.1 M Tris-HCl, pH 8.0, both containing 0.02 M calcium chloride) using a tissue grinder. The homogenates were centrifuged at 9000× *g* for 15 min at 4 °C. The supernatant was considered the intestinal extract (enzymatic preparations). The protein concentration in the intestinal extracts was determined using a previously known protein dosage curve method described by Lowry et al. [45].

##### Determination of Effects of Essential Oil on Activity of Digestive Enzymes and Acetylcholinesterase

Extracts were pre-incubated with the oil at 28 °C for 30 min, and assays were performed in quintuplicate.

α-Amylase activity was measured following the method of Bernfeld [46]. Intestinal extracts (15 μL, 52.5 μg protein) were incubated with 15 μL of oil (0.9–30 ppm) or acetate buffer (control) for 30 min. The reaction with 1% soluble starch in 0.1 M sodium acetate (pH 5.5) containing CaCl_2_ and NaCl was carried out at 50 °C for 10 min, stopped with DNS, heated at 100 °C for 6 min, cooled, and the absorbance was read at 540 nm. Reducing sugars were quantified using a glucose standard curve; one unit of activity corresponded to 1 μmol glucose/min.

Trypsin activity was determined by incubating extracts (15 μL, 200 μg protein) with EO (0.9–30 ppm) and BApNA (Nα-Benzoyl-DL-arginine p-nitroanilide hydrochloride, 8 mM, 5 μL) in 0.1 M Tris-HCl (pH 8.0) at 37 °C for 30 min; Tris buffer was the control. Absorbance was measured at 405 nm, with one unit defined as 1 μmol BApNA hydrolyzed/min. Substrate hydrolysis was validated using bovine trypsin [47].

Acetylcholinesterase (AChE) activity was measured by incubating insect extract (10 μL, 72 μg protein) with EO (0.9–30 ppm), 0.052 M acetylthiocholine, and 0.25 mM DTNB for 3 min at 25 °C. Absorbance at 405 nm was used to quantify thiocholine release, and one unit corresponded to 1 μmol acetylthiocholine hydrolyzed/min [48].

### 4.5. Statistical Analysis

Data were expressed as mean ± standard error and analyzed using GraphPad Prism 8.0. (GraphPad Software, San Diego, CA, USA) Differences among means were evaluated by ANOVA (*p* < 0.05) followed by Tukey’s test (OriginLab, Northampton, MA, USA). Lethal concentrations (LC_10_, LC_50_, LC_90_) and confidence limits were estimated by Probit analysis, and chi-square values with degrees of freedom were calculated using StatPlus Pro 6.2.5.0 (AnalystSoft Inc., Brandon, FL, USA) [27].

## 5. Conclusions

This study demonstrates the significant insecticidal activity of the essential oil (EO) of *M. floribunda* (fumigation: LC_50_ = 3.26 µL/L) and its main compound, (*E*)-caryophyllene (fumigation: LC_50_ = 3.97 µL/L), against the maize weevil. A dose of 62.5 µL/g resulted in significant mortality of *S. zeamais* upon ingestion, while a concentration of 30 µL/g of essential oil compromised the enzymatic activities of amylases and AChE, resulting in nutritional and neurological alterations in the insects. Thus, the EO of *M. floribunda* emerges as a potential ecological alternative to synthetic insecticides in the management of *S. zeamais* in stored maize crops.

## 6. Patents

This work has intellectual property rights and has a national patent application for invention, utility model, certificate of addition of invention and entry into the national phase of the PCT with the National Institute of Industrial Property (INPI) under process number BR1020210213043.

## Figures and Tables

**Table 1:** Relative abundance (%) of constituents of essential oil from *M. floribunda*.

Compound ^a^	Retention Index		% ^d^	S.D. ^e^
	Calculated ^b^	Literature ^c^		
α-pinene	932	931	0.80	0.07
β-pinene	9.74	968	1.01	0.08
myrcene	988	991	0.08	0.01
α-phellandrene	1002	1003	0.35	0.04
α-terpinene	1014	1015	0.03	0.01
ρ-cymene	1020	1023	0.02	0.01
limonene	1024	1027	1.06	0.09
1,8-cineole	1026	1029	3.53	0.35
γ-terpinene	1054	1058	0.18	0.02
linalool	1095	1100	0.27	0.02
bornyl acetate	1284	1287	0.05	0.01
α-cubebene	1348	1351	0.50	0.04
α-ylangene	1373	1373	0.43	0.07
α-copaene	1374	1377	1.37	0.19
sativene	1390	1391	0.12	0.03
α-gurjunene	1409	1411	0.77	0.03
(*E*)-caryophyllene	1417	1422	56.41	2.41
β-gurjunene	1431	1431	1.96	0.28
γ-elemene	1432	1435	0.54	0.09
aromadendrene	1439	1441	0.79	0.12
α-humulene	1452	1455	2.26	0.06
allo-aromadendrene	1458	1463	1.24	0.03
(*E*)-ethyl cinnamate	1465	1468	0.10	0.01
trans-cadina-1(6),4-diene	1475	1475	0.51	0.04
γ-muurolene	1478	1478	0.53	0.02
α-amorphene	1484	1482	0.11	0.01
β-selinene	1489	1488	1.43	0.22
α-selinene	1498	1497	3.85	0.53
α-muurolene	1500	1502	1.88	0.15
δ-amorphene	1511	1509	2.23	0.03
γ-cadinene	1513	1516	1.46	0.02
δ-cadinene	1522	1526	2.09	0.03
α-cadinene	1537	1534	0.32	0.04
α-calacorene	1545	1545	0.35	0.02
germacrene B	1559	1559	0.43	0.04
palustrol	1567	1570	0.32	0.05
globulol	1590	1587	0.73	0.10
viridiflorol	1592	1595	4.02	0.29
ledol	1602	1605	0.67	0.02
α-muurolol	1644	1644	1.76	0.07
β-eudesmol	1649	1653	0.32	0.06
α-cadinol	1652	1658	1.35	0.08
total identified			98.25	

^a^ Constituents listed in elution order in the non-polar DB-5 column. ^b^ Retention index (RI) calculated by retention time in relation to alkane series (C_9_–C_30_) in 30 m DB-5 capillary column. ^c^ Values obtained by Adams (2009) [16]. ^d^ %: area of compound relative to essential oil. ^e^ SD: standard deviation.

**Table 2:** Toxicity by fumigation and lethal concentrations (LC_10_, LC_50_, and LC_90_, µL/L air) of essential oil from *M. floribunda* against *S. zeamais*.

Material	N ^a^	DF ^b^	*X* ^2 c^	LC_10_ ± SE (95%CI) ^d^ (LLC-ULC) ^e^	LC_50_ ± SE (95%CI) ^d^ (LLC-ULC) ^e^	LC_90_ ± SE (95%CI) ^d^ (LLC-ULC) ^e^
*M. floribunda* oil essential	500	3	1.4670	1.89 ± 1.06 (1.64–2.10)	3.26 ± 1.03 (3.06–3.45)	5.63 ± 1.03 (5.27–6.1)
(*E*)-caryophyllene	500	3	2.9388	1.46 ± 1.13 (1.08–1.81)	3.97 ± 1.04 (3.58–4.31)	10.74 ± 1.08 (9.41–12.9)

^a^ Number of insects used in the tests; ^b^ Degrees of freedom; ^c^ Chi-square; ^d^ Lethal concentration (LC); ^e^ Confidence interval for the estimated minimum (LCL) and maximum (UCL) lethal concentrations.

## Data Availability

All data are available in the manuscript itself and in the Supplementary Materials. Any additional questions can be clarified by email to the corresponding author.

## Supplementary Materials

The following supporting information can be downloaded at https://www.mdpi.com/article/10.3390/plants15081272/s1. Table S1: Statistical data from fumigation toxicity bioassays of (E)-caryophyllene; Table S2: ANOVA data from the toxicity tests of *M. floribunda* essential oil by ingestion and fumigation in *S. zeamais*.

## Abbreviations

The following abbreviations are used in this manuscript:

OE	Essential oil
GC/MS	Gas chromatography coupled with mass spectrometry
AChE	Acetylcholinesterase
BApNA	Nα-Benzoyl-DL-arginine p-nitroanilide hydrochloride
*M.*	*Myrciaria*
FDI	Feeding deterrence index
*S.*	*Sitophilus*
RCR	Relative consumption rate
BGR	Biomass gain rate
FCE	Food conversion efficiency
LC_n_	Lethal dose to kill n% of the total insect population (n = 10, 50 or 90%)
ANOVA	Analysis of variance
