# Supplementary material for: Insecticidal Activity of Essential Oil from the Leaves of Myrciaria floribunda (Myrtaceae) Against Sitophilus zeamais in Stored Maize
Source: Plants (Basel). 2026 Apr 21;15(8):1272. doi: 10.3390/plants15081272 (PMC13119956; doi:10.3390/plants15081272)
Supplement: Supplementary file 1 [file plants-15-01272-s001.zip › plants-4241251-supplementary.pdf]

Table S1: Statistical data from fumigation toxicity bioassays of (E)-caryophyllene

	p	n	dose	LCL	UCL	se	chi_square	df	pgof_sig	h	slope	slope_se	slope_sig	intercept	intercept_se	intercept_sig	z	var_m	covariance
1	10	7	1.468710	1.082673	1.815986	1.135985	2.938826	3	0.7094166	1	2.964947	0.2836703	1.433336e-25	-1.776508	0.2098495	2.547706e-17	10.45209	0.967221803	-0.05788171
2	50	7	3.973473	3.584101	4.317214	1.047451	2.938826	3	0.7094166	1	2.964947	0.2836703	1.433336e-25	-1.776508	0.2098495	2.547706e-17	10.45209	0.009926046	-0.05788171
3	90	7	10.749903	9.413123	12.936719	1.081758	2.938826	3	0.7094166	1	2.964947	0.2836703	1.433336e-25	-1.776508	0.2098495	2.547706e-17	10.45209	0.009626124	-0.05788171

Table S2: ANOVA data from the toxicity tests of *M. floribunda* essential oil by ingestion and fumigation in *S. zeamais.*

Paramete	Degrees of freedom	F-statistics	p value
Ingestion assay			
Biomass gain rate	3	49.62	0,002
Relative consumption rat	3	4.14	0.069
Efficiency in conversion of ingested food	3	153.56	0.000
Fumigation assay			
Mortality	3	17.89	0.005
